# Comparative analysis of Methamphetamine and Alcohol-Induced Liver Damage using Ultrasound Shear Wave Elastography

**DOI:** 10.12669/pjms.42.4.6844

**Published:** 2026-04

**Authors:** Ali Mohsin Hasan, Tara Farooq Kareem, Nour Aldeen Sabah Waheed

**Affiliations:** 1 Ali Mohsin Hasan Radiology Department, Al-Qanat Rehabilitation Center, Baghdad, Iraq; 2 Tara Farooq Kareem Consultant Radiologist, Assistant Professor at College of Medicine University of Baghdad, Baghdad, Iraq; 3 Nour Aldeen Sabah Waheed, Radiology Department, Ministry of Health, Baghdad, Iraq

**Keywords:** Alcohol-Induced, Comparative, Elastography, Liver Damage, Methamphetamine, Ultrasound Shear Wave

## Abstract

**Background & Objective:**

Chronic liver disease (CLD) is a significant global health issue, leading to millions of deaths due to complications like cirrhosis and liver cancer. This study utilized shear wave elastography (SWE) to evaluate liver texture in individuals with alcohol and crystal meth addiction. The findings indicate that SWE effectively detects varying stages of liver fibrosis and steatosis, potentially facilitating quicker treatment and better patient outcomes for those at risk of CLD.

**Methodology:**

A prospective study at Al-Qanat Rehabilitation Center December 2023 to May 2024, evaluated liver texture in 315 substance addicts, focusing on liver fibrosis and steatosis. Out of these, 285 patients were analyzed, categorized into alcohol-only, crystal methamphetamine-only, and both substance addicts, while 30 were excluded due to unknown substances. Each patient underwent ultrasound assessments to determine liver fibrosis and steatosis, aiming to correlate addiction type, BMI, and other factors with liver health outcomes.

**Results:**

Study results are substantial. First; Steatosis is more common in alcoholics (56.6% S2) than in crystal methamphetamines (64.3%). Second, crystal meth addicts had more severe fibrosis than alcohol alone addicts compared to alcohol addicts and dual substance (crystal and alcohol) addicts had more fibrosis than alcohol alone or crystal meth alone addicts. Overweight and obese addicts are more likely to have moderate or severe steatosis, although there is no correlation between BMI and fibrosis.

**Conclusion:**

Crystal methamphetamine can cause liver fibrosis without steatosis; this is facilitated by alcohol and obesity.

## INTRODUCTION

Chronic liver disease (CLD) is a major global health issue with progression towards advanced fibrosis, ultimately leading to cirrhosis, hepatic failure and hepatocellular carcinoma. Early recognition and evaluation of fibrosis are paramount because early treatments can slow or even reverse disease progression before the occurrence of serious clinical manifestations. Fibrosis represents an intermediate stage of liver fibrogenesis that leads to cirrhosis, which is differentiated between compensated and decompensated stages in terms of clinical manifestations, with substantial implications for therapeutic interventions and survival.^1^,^2^ Compensated cirrhosis is often asymptomatic and is usually diagnosed incidentally when symptoms of jaundice, ascites, variceal bleeding or hepatic encephalopathy develop.

Patients at this stage usually have a median survival of greater than 12 years, and thus there is considerable therapeutic importance for early detection and observation prior to decompensation.^3^ Although liver biopsy is considered the gold standard for the detection of fibrosis, there are limitations for this methodology. The method is invasive, has risks such as hemorrhage and infection, and is associated with a small but measurable mortality rate. Furthermore, biopsy specimens represent only a small amount of liver tissue and may lead to sampling error, contributing to inaccurate evaluation of disease severity.^4^,^5^ Accordingly, there has been growing interest in noninvasive diagnostic techniques. Shear wave elastography (SWE) is a potentially appealing ultrasonographic method that can access the liver stiffness/attenuation properties related to fibrosis and steatohepatitis. SWE has several advantages, including safety, convenience and cost compared with biopsy^5^ and it demonstrates good accuracy for evaluating fibrosis and steatosis in patients at least with alcohol-related liver disease (ALD).^6^,^7^

The relationship between liver impairment and methamphetamine addiction has been reported recently but is not well documented compared to the drugs related to alcohol-induced hepatotoxicity. Chronic reciprocal abuse of methamphetamine may induce hepatic damage due to the direct toxic effect, hyperthermia, malnourishment, and increased susceptibility to viral infections. As with alcohol abuse, chronic ingestion may result in fibrosis, cirrhosis and other symptoms associated with long-term liver damage. The direct study of SWE in methamphetamine-related liver damage is scarce; however, the elastography assessment of liver stiffness is pertinent from a theoretical perspective to all fibrotic liver diseases. In this way, SWE is likely to provide an important noninvasive tool for characterizing structural hepatic changes within the context of substance use disorders beyond ALD.^8^,^9^

The aim of this study was to investigate the efficacy of alcohol and methamphetamine addiction on the structural status of the liver using shear wave elastography (SWE). By determining liver stiffness in these two dependencies, this study could demonstrate the potential utility of SWE (shear wave elastography) techniques STE (sound touch elastography), USAT (ultrasound attenuation) in detecting early liver fibrosis and steatosis in vulnerable populations at risk for chronic liver disease, so that timely interventions can be provided to achieve better patient outcomes.

## METHODOLOGY

This prospective observational study was conducted from December 2023 to May 2024 at the Al-Qanat Social Rehabilitation Center (part of the Baghdad Medical City complex). A total of 315 patients with drug addiction were admitted. Inclusion criteria were people who drank heavy alcohol (defined as daily drinking for at least one year) or used methamphetamine and drank at least 0.5 grams per day in the past year. Patients who met both of these criteria were also included in the study, making a total of 285 patients included in this study and divided into alcohol only addicts, methamphetamine only addicts and both alcohol and crystalmeth addicts as three groups of drug abusers. Individuals who were heavily dependent on several unknown drugs or herbal medicines other than alcohol and methamphetamine were excluded from the study (30 patients), and patients with end-stage liver failure and advanced fibrosis were also excluded because rehabilitation does not benefit such patients. Therefore, the total number of patients included was 285. Although some patients initially denied the use of certain substances, their substance use was confirmed by clinical examinations and laboratory test results.

### Liver Texture Assessment Using Shear Wave Elastography (SWE) techniques STE (sound touch elastography) for fibrosis and USAT (ultrasound attenuation) for steatosis:

**Fig.1:**
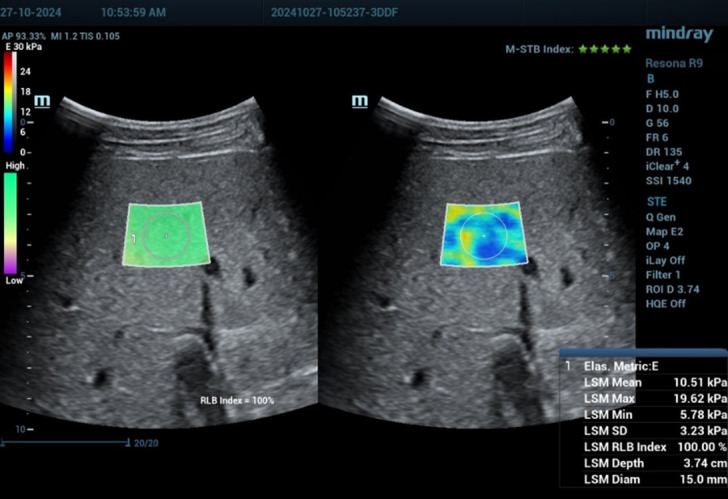
Asymptomatic 24 years old male patient who is addict on crystal methamphetamine (urine test +Ve) for 3 years with elevated liver enzymes. Liver stiffness value obtained by two-dimensional shear wave elastography technique (STE technique by resona R9 ultrasound machine of mindray medical systems) with the mean was 10.5 kPa indicating a significant liver fibrosis with respect to manufacturer reliability and stability index and measurement IQR/median <10. With standardized box positioning.

**Fig.2:**
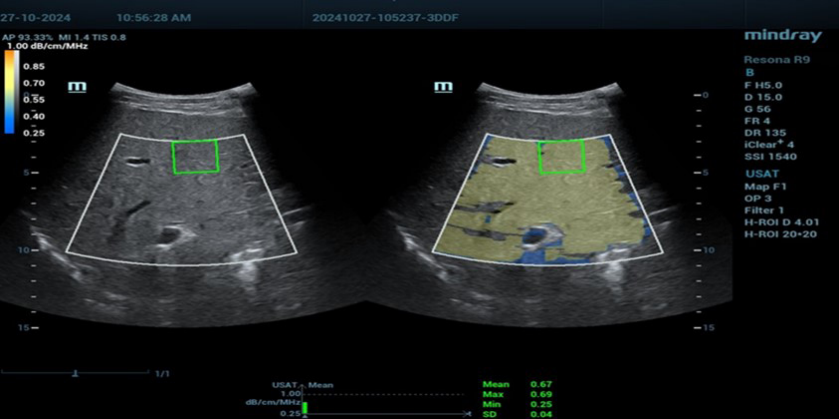
Asymptomatic 25 yr old male patient who is addict on alcohol (urine test +Ve) for 5 years with elevated liver enzymes. Liver attenuation value obtained by two dimensional shear wave elastography technique (USAT technique by resona R9 ultrasound machine of mindray medical systems) with the mean was 0.67 dB/cm/MHz indicating grade 1 liver steatosis with respect to manufacturer reliability and stability index and measurement IQR/median <10. With standardized box positioning.

Liver stiffness and steatosis were assessed using Resona R9 Mindray ultrasound system equipped with a convex single-crystal probe operating at frequencies from 3-9 MHz. For each patient, liver examination was performed at the mid of segment V of the liver with parallel subcostal approach in neutral breath hold, measurement taken approximately 1.5 to 2 cm from the liver capsule, and large blood vessels and bile ducts were avoided to ensure accuracy, with the patient in a supine position, and with their right arm placed above their head to optimize the intercostal space for examination. B-mode ultrasound images were first optimized for qualitative assessment of liver texture, and then liver stiffness was quantitatively measured using SWE. SWE parameters (measured in Kpa for STE) and steatosis parameters (measured in db/cm/MHz for USAT) were each patient was examined twice on the same day by two experienced radiologists with expertise in ultrasound, and the average of each parameter from the two examinations was recorded. The results were compared with the standard cutoff values for staging liver fibrosis and steatosis provided in the manual of the Mindray R9 ultrasound system.^10^,^11^ As in Fig.1 and 2.

### Patient Grouping and Comparison:

Patients were divided into three groups based on their addiction type: (1) Alcohol-only addicts, (2) Crystal methamphetamine-only addicts, and (3) Patients addicted to both alcohol and crystal methamphetamine. A comparative analysis of the stages of liver fibrosis and liver steatosis was conducted across these groups. The correlation between patient characteristics (such as body mass index [BMI], activity level, work style, and duration of addiction) and the degree of liver fibrosis and steatosis at admission was also assessed to identify any significant associations. A p-value of less than 0.05 was considered significant for these associations.

### Ethical approval:

The study was conducted in accordance with the ethical principles that have their origin in the Declaration of Helsinki. It was carried out with patients verbal and analytical approval before sample was taken. The study protocol and the subject information and consent form were reviewed and approved by a local ethics committee according to the document number 292 (including the number and the date in 10/12/2023) to get this approval.

## RESULTS

**Fig.3:**
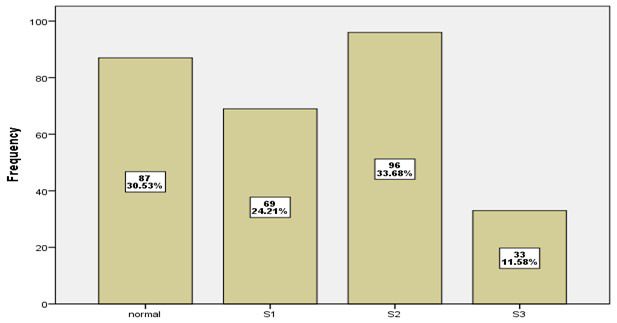
distribution of patients according to Steatosis Degree.

A breakdown of the study population according to key demographic and clinical variables including age groups, gender, BMI, and addiction duration is provided in Table-I. Age Groups: The majority of patients fall into the 20-29 (38.6%) and 30-39 (36.8%) age brackets, while the youngest <20 years) and oldest (≥60 years) groups account for a small portion of the study population (8.4% and 1.8%, respectively). Gender: The sample is overwhelmingly male (95.1%), with females representing only 4.9% of the population. BMI: Most patients have a normal BMI (55.1%), but a significant number are either overweight (37.2%) or obese (7.7%). Duration of Addiction: The largest group of patients (47.0%) have been addicted for more than five years, while about one-third (33.7%) have been addicted for less than three years, and 19.3% for three to five years. Fig.3 show that 33.68% of patients have Steatosis Degree (2), while 24.21% of patients have Steatosis Degree (1) and only 11.58% of patients have Steatosis Degree (3). Normal liver in 30.53% of patients.

**Table-I:** distribution of patients according to (age groups, gender, BMI, Duration of addict).

Variables		Frequency	Percentage
Age groups (years)	<20	24	8.4
	20-29	110	38.6
	30-39	105	36.8
	40-49	33	11.6
	50-59	8	2.8
	≥60	5	1.8
Gender	Male	271	95.1
	Female	14	4.9
BMI	Normal	157	55.1
	Overweight	106	37.2
	Obese	22	7.7
Duration of addict (years)	<3	96	33.7
	3-5	55	19.3
	>5	134	47.0
Total		285	100

**Fig.4:**
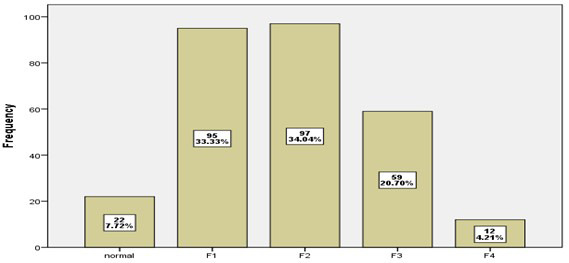
distribution of patients according to Fibrosis Degree.

**Fig.5:**
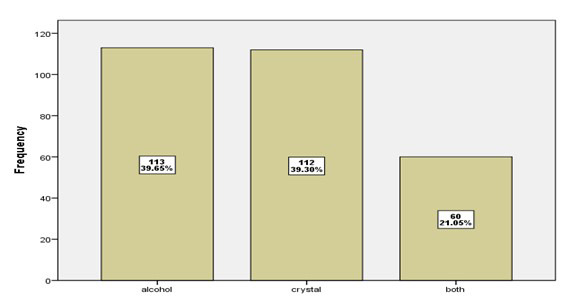
distribution of patients according to substance used.

Fig.4 show that 34.04% of patients have fibrosis Degree (2), while 33.33% of patients have fibrosis Degree (1), 20.7% of patients have Steatosis Degree (3). and only 4.21% of patients have fibrosis Degree (4). Normal liver in 7.72% of patients. Fig.5 showed that 39.65% of patients addicted on alcohol, while 39.3% of them addicted on crystal and 21.05% of patients addicted on both (alcohol and crystal).

The relationship between substance intake types (Alcohol, Crystal, and Both) and various demographic factors is shown in Table-II. Alcohol users are predominantly aged 30-39 (54.0%) and mostly male (97.3%), with a significant duration of addiction over five years (87.6%). Crystal users are primarily aged 20-29 (50.9%) and also male (92.0%), with many having less than three years of addiction (61.6%). Users of both substances are mainly in the 20-29 age group (55.0%) and predominantly male (96.7%). In terms of BMI, alcohol users show a higher percentage of overweight individuals (54.0%), while crystal users have a majority with a normal BMI (72.3%). Significant associations exist between substance types and age, duration of addiction, and BMI.

**Table-II:** Association between Substances intake types and (age groups, gender, BMI, Duration of addict).

Substances intake types					
Age groups years		*Alcohol*	*Crystal*	*Both*	*P-value*
	<20	1 (0.9%)	17 (15.2%)	6 (10.0%)	
	20-29	20 (17.7%)	57 (50.9%)	33 (55.0%)	0.0001
	30-39	61 (54.0%)	26 (23.2%)	18 (30.0%)	
	40-49	22 (19.5%)	8 (7.1%)	3 (5.0%)	
	50-59	6 (5.3%)	2 (1.8%)	0 (0.0%)	
	≥60	3 (2.7%)	2 (1.8%)	0 (0.0%)	
Gender	Male	110 (97.3%)	103 (92.0%)	58 (96.7%)	
	Female	3 (2.7%)	9 (8.0%)	2 (3.3%)	0.14
Duration of addiction (years)	<3	5 (4.4%)	69 (61.6%)	22 (36.7%)	
	3-5	9 (8.0%)	25 (22.3%)	21 (35.0%)	0.0001
	>5	99 (87.6%)	18 (16.1%)	17 (28.3%)	
	Normal	42 (37.2%)	81 (72.3%)	34 (56.7%)	
BMI	Overweight	61 (54.0%)	26 (23.2%)	19 (31.7%)	0.0001
	Obese	10 (8.8%)	5 (4.5%)	7 (11.7%)	
Total		113 (100%)	112 (100%)	60 (100%)	

The relationship between substance intake types (Alcohol, Crystal, and Both) and the degrees of steatosis and fibrosis is highlighted in Table-III. Alcohol users predominantly fall into the S2 steatosis category (56.6%) and show higher fibrosis levels, particularly in F1 (36.3%). In contrast, crystal users mostly have normal steatosis (64.3%) and a significant portion in F2 fibrosis (37.5%). Users of both substances exhibit severe liver damage, with notable steatosis and fibrosis levels, while crystal users generally show less severe conditions.

**Table-III:** Association between Substances intake types and (Steatosis, fibrosis degree).

Substances intake types					
		Alcohol	Crystal	Both	P-value
Steatosis Degree	Normal	5 (4.4%)	72 (64.3%)	10 (16.7%)	
	S1	18 (15.9%)	34 (30.4%)	17 (28.3%)	0.0001
	S2	64 (56.6%)	5 (4.5%)	27 (45.0%)	
	S3	26 (23.0%)	1 (0.9%)	6 (10.0%)	
Fibrosis Degree	Normal	15 (13.3%)	4 (3.6%)	3 (5.0%)	
	F1	41 (36.3%)	35 (31.3%)	19 (31.7%)	0.017
	F2	29 (25.7%)	42 (37.5%)	26 (43.3%)	
	F3	20 (17.7%)	29 (25.9%)	10 (16.7%)	
	F4	8 (7.1%)	2 (1.8%)	2 (3.3%)	
Total		113 (100%)	112 (100%)	60 (100%)	

The analysis of BMI categories reveals a significant correlation between higher BMI and the severity of steatosis, with normal BMI patients showing the highest percentage of normal steatosis (47.8%) and the lowest percentage of severe steatosis (4.5%) is provided in Table-IV. In contrast, obese patients exhibit the highest rates of moderate (36.4%) and severe steatosis (31.8%). However, the association between BMI and fibrosis severity is not significant, although obese individuals have a slightly higher incidence of cirrhosis (9.1%) compared to those with normal or overweight BMIs.

**Table-IV:** Association between BMI and steatosis and fibrosis degrees.

BMI					
		Normal	Overweight	Obese	P-value
Steatosis Degree	Normal	75 (47.8%)	11 (10.4%)	1 (4.5%)	
	S1	37 (23.6%)	26 (24.5%)	6 (27.3%)	0.0001
	S2	38 (24.2%)	50 (47.2%)	8 (36.4%)	
	S3	7 (4.5%)	19 (17.9%)	7 (31.8%)	
Fibrosis Degree	Normal	14 (8.9%)	7 (6.6%)	1 (4.5%)	
	F1	55 (35.0%)	33 (31.1%)	7 (31.8%)	0.5
	F2	57 (36.3%)	33 (31.1%)	7 (31.8%)	
	F3	28 (17.8%)	26 (24.5%)	5 (22.7%)	
	F4	3 (1.9%)	7 (6.6%)	2 (9.1%)	
Total		157 (100%)	106 (100%)	22 (100%)	

## DISCUSSION

The results of this study are the new and up to date discovery of the effect of crystal methamphetamine as a direct causative highly addictive drug for liver fibrosis and evaluating this fibrosis quantitatively using new ultrasound technology with accurate diagnosis for quick clinical management to be undertaken. It also highlight important patterns in liver steatosis and fibrosis in individuals who abuse alcohol and crystal methamphetamine. Degree-2 steatosis (moderate) was the most frequent pattern, followed by Degrees 1 and 3 but normal liver results were observed in nearly one-third of the patients, indicating that changes related to fatty liver are common but not generally pronounced. Most cases of fibrosis were moderate with a higher proportion of Degree-2 and first Degree; however, advanced fibrosis was rare. This distribution reflects the dominance of early patients or newly diagnosed liver disease and is in keeping with previous studies showing that initial fibrosis stages are more frequent in alcohol-induced liver disease and substance-related hepatic damage.^12^,^13^ Prevalence’s of addictive patterns were indistinguishable for use of alcohol, crystal methamphetamine and dual substances. Simultaneous exposure appeared to aggravate the liver’s pathology, consistent with previous research that demonstrate polydrug use accelerate liver damage and escalate disease severity.^14^,^15^

There were significant associations between the type of substance, patient’s age, addiction duration and BMI but not gender. OR for alcoholism was higher in 30-39 age group, whereas current crystal methamphetamine use was higher in younger adults which is consistent with stereotype of previous substance use pattern.^16^,^17^ The high burden of substance use problems in men is congruent with existing epidemiological evidence.^18^ Alcohol dependency generally lasts longer than crystal use, which could be suggestive of the more imminent health consequences resulting from meth exposure.^19^ Differences in BMI distribution were evident consistent with those previously described, where alcohol consumers are more likely to be overweight or obese while methamphetamine users are typically normal weight, indicating contrasting metabolic effects of these substances.^20^ Liver outcomes analysis showed that alcohol consumers had higher scores of steatosis, in moderate and severe grades especially, supporting previous findings on the relationship between chronic alcohol ingestion and hepatic fat accumulation.^21^

In contrast, the most of crystal methamphetamine users had normal steatosis, which suggests that no fat accumulates and methamphetamine toxicity may present more frequently as fibrosis and inflammation than fat deposition.^22^ Both alcohol and polysubstance users had more fibrosis than non-users, suggesting an additive inflammatory effect of alcohol on liver.^23^ On the other hand, in methamphetamine users, mainly moderate degree of fibrosis was observed while only few exhibited advanced cases.^24^ The composite category of dual use along with alcohol use is associated with a more severe degree of liver injury compared to methamphetamine used alone. A strong correlation was observed between Body Mass Index (BMI) and the grade of steatosis, supporting the assertion that obese individuals are more likely to have moderate to severe fatty liver thus confirming previous evidence that has linked obesity with the progression of non-alcoholic fatty liver disease (NAFLD).^25^,^26^ However, BMI was not found to be related with fibrosis, thus body weight per se might less determine the emergence of fibrosis than metabolic factors.^27^ Moreover, more advanced fibrosis in obese than nonobese patients is consistent with studies linking obesity to a progression to cirrhosis.^28^

### Strength and Limitations:

Shear wave elastography is a noninvasive and valid method for the assessment of liver fibrosis and steatosis in a large population of substance-dependent patients, which provides clinically useful comparisons between alcohol and methamphetamine users. Nevertheless, we were not able to draw causal inferences from our cross-sectional research design and not taking into account advanced liver failure may have underestimated the prevalence of severe disease. Male predominance and absence of histological verification might restrict generalizability and diagnostic accuracy.

## CONCLUSION

Methamphetamine usage can cause liver fibrosis without steatosis. Alcohol, obesity, and a sedentary lifestyle increase liver fibrosis, especially in people working less than 8 hours a day. The length of drug abuse promotes liver damage. Steatosis and fibrosis make liver function harder to repair in obese people. Significantly, such organized programs reduce activity less among obese, elderly, or sedentary people. Steatosis of the liver is easier to reverse than fibrosis, and young individuals recover faster. Combining methamphetamine with alcohol, obesity, age, and sedentary lifestyle is co-morbidity.

### List of Abbreviations:

**ALD:** Alcohol-Related Liver Disease

**BMI:** Body Mass Index

**CLD:** Chronic Liver Disease

**dB/cm/MHz:** Decibel per Centimeter per Megahertz

**F1–F4:** Fibrosis Stages (Stage 1–Stage 4)

**IQR:** Interquartile Range

**kPa:** Kilopascal

**NAFLD:** Non-Alcoholic Fatty Liver Disease

**NASH:** Non-Alcoholic Steatohepatitis

**STE:** Sound Touch Elastography Technique (for fibrosis assessment)

**SWE:** Shear Wave Elastography

**S1–S3:** Steatosis Grades (Grade 1–Grade 3)

**USAT:** Ultrasound Attenuation Technique (for steatosis assessment)
